# A versatile photodetector assisted by photovoltaic and bolometric effects

**DOI:** 10.1038/s41377-020-00396-3

**Published:** 2020-09-10

**Authors:** Wei Jiang, Tan Zheng, Binmin Wu, Hanxue Jiao, Xudong Wang, Yan Chen, Xiaoyu Zhang, Meng Peng, Hailu Wang, Tie Lin, Hong Shen, Jun Ge, Weida Hu, Xiaofeng Xu, Xiangjian Meng, Junhao Chu, Jianlu Wang

**Affiliations:** 1 State Key Laboratory of Infrared Physics, Shanghai Institute of Technical Physics, Chinese Academy of Sciences, 500 Yutian Road, Shanghai, 200083 China; 2 University of Chinese Academy of Sciences, Beijing, 100049 China; 3 Department of Applied Physics, Donghua University, No. 2999, North Renmin Road, Songjiang District, Shanghai, 201620 China

**Keywords:** Imaging and sensing, Optoelectronic devices and components

## Abstract

The advent of low-dimensional materials with peculiar structure and superb band properties provides a new canonical form for the development of photodetectors. However, the limited exploitation of basic properties makes it difficult for devices to stand out. Here, we demonstrate a hybrid heterostructure with ultrathin vanadium dioxide film and molybdenum ditelluride nanoflake. Vanadium dioxide is a classical semiconductor with a narrow bandgap, a high temperature coefficient of resistance, and phase transformation. Molybdenum ditelluride, a typical two-dimensional material, is often used to construct optoelectronic devices. The heterostructure can realize three different functional modes: (i) the p–n junction exhibits ultrasensitive detection (450 nm–2 μm) with a dark current down to 0.2 pA and a response time of 17 μs, (ii) the Schottky junction works stably under extreme conditions such as a high temperature of 400 K, and (iii) the bolometer shows ultrabroad spectrum detection exceeding 10 μm. The flexible switching between the three modes makes the heterostructure a potential candidate for next-generation photodetectors from visible to longwave infrared radiation (LWIR). This type of photodetector combines versatile detection modes, shedding light on the hybrid application of novel and traditional materials, and is a prototype of advanced optoelectronic devices.

## Optoelectronics: lighting the way to advanced photodetectors

Scientists from China have made photodetectors from semiconducting and two-dimensional materials that could lay the foundations for advanced optoelectronic devices. Photodetectors made from two-dimensional semiconducting materials such as quantum dots, nanowires, nanoflakes, and nanofilms have led to smaller devices that exhibit ultrafast response times, exceptional sensitivity, or a broad-spectrum response compared to devices made from traditional materials. Currently, however, no device can combine all these attributes. A team of Chinese researchers led by Jianlu Wang from the Shanghai Institute of Technical Physics has now developed a hybrid photodetector made from an ultrathin film of vanadium dioxide and a molybdenum telluride nanoflake. The photodetector combines a rapid response with high sensitivity and is capable of detecting visible to longwave infrared radiation, paving the way for better-performing devices for use in a range of optoelectronic applications.

## Introduction

Photodetectors^1^, due to their widespread application and superior status, have emerged as a research hotspot since their appearance in the early 1910s. According to the energy conversion process, photodetectors can generally be categorized as quantum detectors and thermal detectors^1–4^. Quantum detectors can directly convert photons into electric signals, while thermal detectors generate electric output through changes in physical properties under incident radiation. Depending on the detection mechanism, the class of photodetectors can be further divided into different types, including photoconductive detectors (PbSe, PbS, HgCdTe, InSb), photovoltaic detectors (Si, Ge, HgCdTe, InAs, InSb), and quantum well detectors (GaAs/AlGaAs). The thermal detectors also include thermopiles (Pt-Ir), bolometers (VO_2_, α-Si), and pyroelectric detectors (LiTaO_3_, PVDF)^2,3,5,6^. Recently, the emergence of detectors based on low-dimensional materials^7–12^, such as quantum dots, nanowires, nanoflakes, and nanofilms, has led to a boom in the development of excellent photodetectors. Nanostructured materials with reduced physical size and unique band structure bring about incredible performance compared to traditional materials. In particular, two-dimensional (2D) semiconductors have received more attention for their plane characteristics and lack of dangling bonds^13^, which is an apparent advantage for building various vertical heterostructures^14^ and integration with CMOS^15^. In fact, promising progress has been made in the development of 2D photodetectors^16,17^. Photodetectors based on graphene^18^, transition metal chalcogenides (TMDCs)^19^, and black phosphorus^20^ exhibit ultrafast responses, exceptional sensitivities, or broad-spectrum responses. Localized field modulation^21,22^ and heterojunction construction^23,24^ are usually applied to enhance various detector performances. However, there is no evidence of a detector with excellent comprehensive performance. According to the demands for next-generation infrared detectors^2^, can we develop an uncooled versatile detector with rapid response, high sensitivity, and broadband response?

Here, a new concept of a photodetector combining photovoltaic detectors with a bolometer is demonstrated. A photon with energy larger than the bandgap can be directly absorbed by an electron in the valence band. Then, the electron undergoes an optical transition to the conductance band. An electron–hole pair will be generated after the transition and separated by a built-in electric field or a bias voltage, which is the origin of the photocurrent of the photovoltaic detector. Due to the rapid separation of electron–hole pairs, the photovoltaic detector is known for its sensitivity, despite its limited response range. When the energy of mid-infrared or far-infrared radiation is not sufficient to allow the electron to undergo an optical transition, electron–phonon interactions occur between phonons and low-energy photons. Therefore, the thermal equilibrium and carrier density are disturbed, causing fluctuations in resistance. This is the so-called bolometric effect, which is particularly significant in materials with a high temperature coefficient of resistance, such as VO_2_^25^. Compared to the photovoltaic detector, the bolometer is not wavelength selective, but the response rate is relatively slow. To combine the advantages of the two detector types, a heterostructure device based on VO_2_ and MoTe_2_ is fabricated. There are three operation modes integrated into one detector, including the p–n junction mode, Schottky junction mode, and bolometer mode. The three modes can be flexibly switched between for different situations. This detector achieves detection from visible light to longwave infrared radiation (LWIR) and simultaneously exhibits good performance in terms of a rapid response and a high sensitivity.

## Results

### Device structure and characterization

**Fig. 1:**
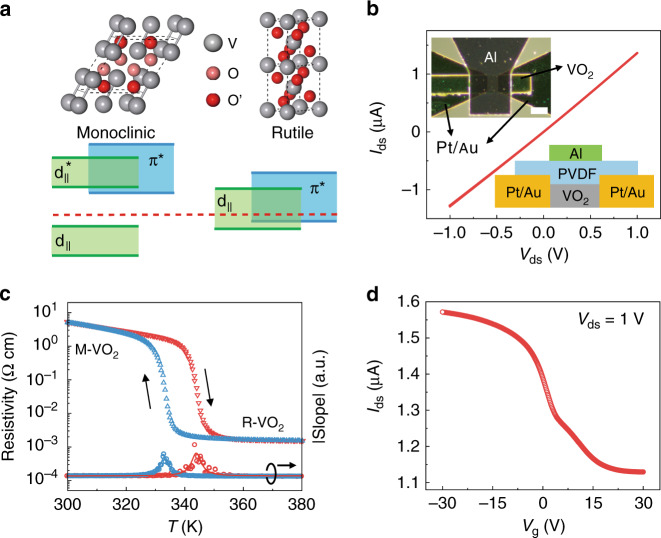
Structure and electronic characterization of VO_2_. **a** Schematic of the crystal structure and band structure of monoclinic and rutile VO_2_. **b** Output characteristics of the P(VDF-TrFE)-gated VO_2_ FET; the insets show the schematic and corresponding optical image. Scale bar, 20 μm. **c** Resistivity of the VO_2_ film measured under vacuum as a function of temperature (red, heating; blue, cooling). The lower points and fitted curves show the abs(slope) of the resistivity. **d** Transfer characteristics at *V*_ds_ = 1 V of the VO_2_ FET.

VO_2_^25–27^ is a classic phase transition material with a metal–insulator transition (MIT) near room temperature (340 K). When the temperature is below the transformation temperature (*T*_*c*_), VO_2_ is in a monoclinic phase^28^. The O atoms are in different chemical states, as labeled in Fig. 1a. In this phase, the Fermi level is in the middle of the *d*_||_ bonding orbital and *d*_||_^*^ antibonding orbital^29^, indicating that VO_2_ is a semiconductor. When the temperature is above *T*_*c*_, VO_2_ transforms to the rutile phase, and the O atoms are in the same chemical state. In this phase, the Fermi level passes through the overlap zone of the *d*_||_ orbital and π^*^ orbital. As a result, VO_2_ shows metallic behavior.

Here, the VO_2_ film is synthesized on a sapphire substrate (see “Materials and methods”). The thickness is measured to be ~22 nm with a surface undulation of <2 nm (Fig. S1). As shown in Fig. S2a, X-ray diffraction (XRD) and Raman spectroscopy are performed to identify the phase and purity. The peaks marked (002) and ($\bar 4$02) in Fig. S2a result from the monoclinic phase VO_2_^30^. Raman peaks are distinguished in Fig. S2b, but all of them can be ascribed to the vibration modes of VO_2_^31^, indicating that the sample is a high-quality VO_2_ film with no other vanadium oxides. First, the basic electrical properties of VO_2_ are investigated by using a top-gated field-effect transistor (FET) with poly(vinylidene fluoride-trifluoroethylene) (P(VDF-TrFE)) as a dielectric layer, as shown in Fig. 1b. The output curve in Fig. 1b indicates that the Pt/Au electrode can form good ohmic contact with VO_2_. As shown in Fig. 1c, the resistivity changes by more than 10^3^ between monoclinic VO_2_ (M-VO_2_) and rutile VO_2_ (R-VO_2_), showing a significant relationship with temperature. A hysteresis phenomenon appears during thermal cycling. The phase transition temperature is ~344 K during the heating process and ~333 K during the cooling process, extracted from the slope of the temperature-dependent resistivity curve. The temperature of the semiconductor to metal transformation is slightly higher than 340 K because of the strain^32^ between domains as well as the inhomogeneous strain fields with the substrate^33^. Figure 1d shows the transfer characteristic curve. The VO_2_ film behaves as an unusual p-type material, but this behavior can be achieved by adjusting the temperature in the synthesis process^34^.

**Fig. 2:**
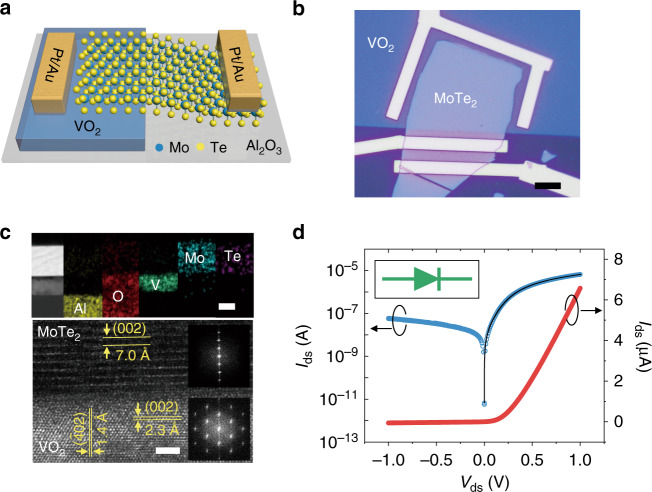
Structure and properties of the heterostructure. **a**, **b** Schematic and optical micrograph of the VO_2_/MoTe_2_ heterostructure on a sapphire substrate. Scale bar, 10 μm. **c** Cross-sectional TEM image and corresponding EDS mappings of the heterostructure. Scale bar, 20 nm (top); an HRTEM image of the interface of the heterostructure is shown at the bottom. The insets show the corresponding fast Fourier transform patterns indexed to MoTe_2_ and VO_2_. Scale bar, 2 nm (bottom). **d** Linear plots (red) and logarithmic plots (blue) of *I*_ds_ as a function of *V*_ds_. The inset symbol represents the junction mode.

Figure 2a shows a schematic of our heterostructure. A thick MoTe_2_ flake is chosen to fabricate a heterostructure with VO_2_ because it always behaves as an n-type material^35^ and its bandgap is relatively narrow compared to other TMDCs^16^. Figure 2b shows an optical image of the fabricated heterostructure. The thickness of MoTe_2_ is identified by AFM to be ~40 nm (Fig. S3a). Figure S3b presents the Raman modes of the typical thick MoTe_2_, in which the intensity of the A_1g_ vibration is much weaker than that of the E_2g_ vibration mode^36^. Finally, P(VDF-TrFE) is spin-coated on the device to protect it from oxygen. With such optimization, a heterostructure between VO_2_ and MoTe_2_ is achieved. A cross-sectional TEM image and the corresponding energy dispersive X-ray spectroscopy (EDS) mappings of the MoTe_2_/VO_2_ heterostructure are shown at the top of Fig. 2c. Each component is identified and marked in the images. From left to right, they are Al, O, V, Mo, and Te. This heterostructure is a tightly stacked structure with almost no impurity layer observed. The detailed atomic structures of VO_2_ and MoTe_2_ are investigated by high-resolution TEM, as shown at the bottom of Fig. 2c. The two insets are fast Fourier transform patterns of the VO_2_ and MoTe_2_ data. MoTe_2_ has a clear layered structure, and the layer-to-layer spacing is ~0.70 nm^37^. VO_2_ also shows high crystallinity, and the *d*-spacings of (002) and ($\bar 4$02) are 0.23 and 0.14 nm^38^, respectively, corresponding to the XRD pattern.

Figure 2d shows the output characteristics of this device. The classical diode output curve (red) proves the existence of a junction. A rectification factor of 10^2^ can be achieved, shown on a logarithmic scale (blue curve). The curve is fitted by the following p–n junction diode equation including a series resistance *R*_*s*_^39^.

$${\it{I}}_{{\it{ds}}} = \frac{{{\it{nV}}_{\it{T}}}}{{{\it{R}}_{\it{s}}}}{\it{W}}\left[ {\frac{{{\it{I}}_0{\it{R}}_{\it{s}}}}{{{\it{nV}}_{\it{T}}}}\exp \left( {\frac{{{\it{V}}_{{\it{ds}}} + I_0R_s}}{{{\it{nV}}_{\it{T}}}}} \right)} \right] - {\it{I}}_0,$$

in which *V*_*T*_ *=* *k*_*b*_*T/e* is the thermal voltage at temperature K, *k*_B_ is the Boltzmann constant, *e* is the electron charge, *I*_0_ is the reverse bias current, *n* is the ideality factor, and *W* is the Lambert *W* function. In this heterostructure, *n* is fitted as 2.2 (for an ideal p–n junction, *n* = 1) with an *R*_*s*_ of 0.99 MΩ, indicating that the transport characteristics are dominated by recombination, probably because of the large density of surface trap states from the polycrystalline VO_2_ film acting as recombination centers^40^. To further prove that a junction is generated between VO_2_ and MoTe_2_, the contact and electrical transport properties of fractional VO_2_ and MoTe_2_ are measured and presented in Fig. S4. As shown in Fig. S4a, b, ohmic contact is simultaneously achieved for VO_2_ and MoTe_2_, which means that the junction behavior only results from the VO_2_/MoTe_2_ heterojunction part. Furthermore, to prove that it is a p–n junction, the transfer characteristics of MoTe_2_ are measured in a P(VDF-TrFE)-gated FET. Graphene is transferred as a top electrode (Fig. S4c). P(VDF-TrFE) is not only a protective membrane but also a powerful dielectric layer with a strong polarization electric field^41^. As shown in Fig. S4d, when the gate voltage (*V*_*g*_) is switched from −30 to 30 V, the current between the drain and source (*I*_ds_) increases logarithmically, conforming to the n-type behavior of thick MoTe_2_. As VO_2_ is p-type and thick MoTe_2_ has n-type characteristics, the heterostructure is a typical p–n junction with a built-in electric field directed from MoTe_2_ to VO_2_.

### Photoresponse in the p–n junction mode

**Fig. 3:**
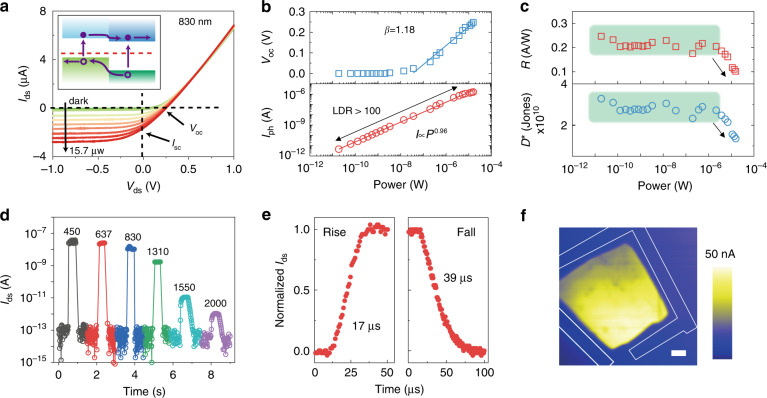
Power and wavelength dependence of the p–n junction mode. **a** *I*_ds_ − *V*_ds_ curves for incident laser powers from 0 to 15.7 μW. The inset shows a schematic of the photovoltaic mechanism for detecting visible to near-infrared radiation. **b** *V*_OC_ and *I*_ph_ as a function of the incident laser power. **c** Responsivity and detectivity as a function of the incident laser power. **d** Temporal response of the photocurrent under laser illumination of 450, 637, 830, 1310, 1550, and 2000 nm; all the powers are fixed at 80 nW. **e** Rise and fall times of the normalized photocurrent under zero bias measured by an oscilloscope. **f** Mapping of the photocurrent measured under scanning laser illumination at 830 nm; the yellow area corresponds to the overlap of the heterostructure. Scale bar, 5 μm.

Figure 3a shows the output characteristics under 830 nm illumination of various laser powers. The open-circuit voltage (*V*_oc_) and short-circuit current (*I*_sc_) increase as the power increases from 0 to 15.7 μW. Due to the photovoltaic effect, the electron–hole pairs generated by electron transition are separated by the built-in electric field and collected by the electrodes (inset of Fig. 3a). Herein, |*I*_ds_| increases to ~2 μA at zero bias under 15.7-μW illumination, which is several orders of magnitude higher than the dark current of 2 pA. To investigate the power dependence of our device, *V*_oc_ and *I*_ph_ (*I*_ph_ = *I*_dark_ − *I*_sc_) for various powers are plotted and fitted in Fig. 3b. *V*_oc_ increases with power, excluding the small values outside of the experimental voltage resolution. *V*_oc_ can be described by the following equation^42^:

$$\frac{{{\it{dV}}_{{\it{\mathrm{oc}}}}}}{{{\it{d{\mathrm{log}}}}\left( P \right)}} = 2.3 \times \frac{2}{\beta }\frac{{k_bT}}{e},$$

where *β* is the recombination order. *β* = 1 represents Shockley–Read–Hall (SRH) recombination, and *β* = 2 indicates Langevin recombination. The experimental data are fitted, and *β* is evaluated as 1.18, indicating that the SRH (monomolecular) process dominated recombination. In other words, a large density of the charge-trap states of MoTe_2_ or VO_2_ participated in recombination. *I*_ph_ shows a linear dependence on power, complying with the power law *I*_ph_ = *P*^*α*^, where *α* is calculated to be 0.96. This value is very close to the ideal value, 1, indicating an efficient process of electron–hole pair generation and recombination. The linear dynamic range of this device is >100, exceeding that of most 2D material-based heterostructures^42,43^. The output electric power *P*_el_ = *I*_ds_*V*_ds_ as a function of *V*_ds_ is plotted in Fig. S5a. The fill factor FF = *P*_el, max_/(*I*_sc_*V*_oc_) (*P*_el, max_ is the maximum power point in Fig. S5a) and the power conversion efficiency *η* = *P*_el, max_/*P* are extracted and plotted in Fig. S5b, c. It is shown that FF is maintained between 0.24 and 0.32 under different incident laser powers, and the value of *η* is ~0.90% on average. Figure 3c shows the power-dependent responsivity (*R*) and detectivity (D^*^)^43^ of our heterostructure, which are defined as:

$${\it{R}} = \frac{{{\it{I}}_{{\it{\mathrm{ph}}}}}}{{\it{P}}},$$

$${\it{D}}^ \ast = \frac{{{\it{R}}\sqrt {\it{S}} }}{{\sqrt {2eI_{{\it{\mathrm{dark}}}}} }}.$$

*S* is the overlapped area of the heterostructure (1133 μm^2^), *e* is the electron charge, and *I*_dark_ is the dark current, which we evaluated as 0.2 pA. Here, *R* is calculated to be 0.22 A/W on average before absorbance saturation and decreases to 0.1 A/W after saturation. D^*^ shows the same tendency as *R*, and the average of D^*^ is ~3 × 10^10^ Jones. The external quantum efficiency (EQE) is the ratio of the number of collected carriers to the number of incident photons, determined by the equation EQE = *Rh*c/*λe*, where *λ* is the wavelength of light, *h* is Planck’s constant, and *c* is the speed of light. The EQE at 830 nm is ~30% before saturation (Fig. S5d), comparable to that of reported 2D p–n junctions^44^.

Figure 3d exhibits the rapid temporal response at different wavelengths (*λ*) with a fixed power of 80 nW. The photocurrent shows a remarkable and rapid change between dark state and laser illumination, even though the on/off ratio decreases under near-infrared radiation. However, the heterostructure still behaves excellently under 2 μm laser illumination with an on/off ratio of more than 10, which is incredible for most 2D p–n diodes^45,46^. The responsivity for various wavelengths is calculated and presented in Fig. S6a. The device shows a broadband response of the photovoltaic effect from 0.45 to 2 μm. A rapid decrease occurs when the wavelength ranges from 1.31 to 2 μm because the bandgap of bulk MoTe_2_ is ~0.9 eV and only VO_2_ contributes to the responsivity when *λ* is longer than 1.31 μm. In addition, the cutoff *λ* here is 2 μm, very close to the bandgap of VO_2_, estimated from the absorbance spectrum in Fig. S6b. As the response time exceeds the limit of the photoelectric measurement system, an oscilloscope is introduced to measure the rise and fall times of the photocurrent under zero bias. As shown in Fig. 3e, the rise and fall times are evaluated as 17 and 39 μs, respectively, which are quicker than those of other MoTe_2_- or VO_2_-based heterostructures^43,47^ as a result of rapid separation and recombination. However, the response time here is also limited by the preamplifier or oscilloscope^48^, and it may be faster. The response times for other wavelengths are shown in Fig. S6c. The response rate becomes slower when the wavelength exceeds 1.31 μm, ascribed to the influence of the bolometric effect. Scanning photocurrent microscopy is an effective method to identify the area where photocurrent is generated. *I*_ds_ at zero bias is measured as a laser spot scans the surface of the heterostructure. Figure 3f shows the mapping of the measured photocurrent of this p–n junction, in which the bright yellow area represents the area where spontaneous charges are separated. It is clear that the area is the overlap of MoTe_2_ and VO_2_, further proving that the photoresponse is dominated by the photovoltaic effect from the MoTe_2_/VO_2_ junction. Photocurrent mappings for other wavelengths and devices are shown in Figs. S7 and S8, proving the repeatability and high performance of our devices.

### Bolometer mode

**Fig. 4:**
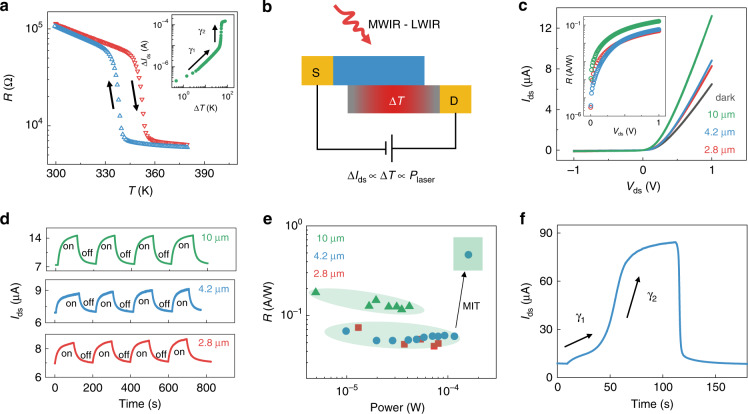
Bolometer mode of the heterostructure. **a** Resistance of the VO_2_/MoTe_2_ heterostructure as a function of temperature (red, heating; blue, cooling). The inset shows the extracted Δ*I*_ds_ and Δ*T*, in which Δ*I*_ds_ increases with Δ*T* with slopes of *γ*_1_ and *γ*_2_. **b** Schematic of the bolometer mode for detecting MWIR to LWIR. **c** *I*_ds_ − *V*_ds_ curves for different incident laser wavelengths (black, dark; green, 10 μm, 41.6 μW; blue, 4.2 μm, 40.6 μW; red, 2.8 μm, 37.1 μW). The inset shows the calculated responsivity for the three wavelengths as a function of *V*_ds_. **d** Temporal response of the photocurrent under laser illumination of 10 μm, 4.2 μm, and 2.8 μm. **e** Power dependence of the heterostructure. The responsivity rapidly increases when the laser power exceeds 157 μW, indicating the metal–insulator transformation. **f** Temporal response of the photocurrent under high-power laser illumination of 157 μW.

The photovoltaic detector is a device that works under zero or negative bias, but here, the p–n junction under forward bias can also be used. When the forward bias is larger than the built-in electric field, the p–n junction can be considered a resistor as the SCR of the p–n junction disappears. The device transforms into a bolometer because of the presence of VO_2_. First, *I*_ds_ at *V*_ds_ = 1 V during thermal cycling is measured from 300 to 380 K and back to 300 K. As shown in Fig. 4a, the resistance of this heterostructure is calculated and plotted as a function of temperature. The resistance shows a rapid decrease with increasing temperature, especially in the MIT range. Therefore, our device is capable of infrared detection similar to the VO_2_ bolometer. As the detection of infrared radiation is a process of decreasing resistance, *ΔI* and *ΔT* are extracted and shown in the inset of Fig. 4a. It is clear that *ΔI* increases with a slope of *γ*_1_ before the MIT and increases rapidly during the MIT with a slope of *γ*_2,_ in which *γ*_2_ > *γ*_1_. Figure 4b shows the infrared detection mechanism of our device. When under laser illumination below energy causing the MIT, the carrier density of VO_2_ changes after absorbing energy from mid-wavelength infrared radiation (MWIR) to LWIR. The increased carriers drift to opposite electrodes in the external electric field, resulting in a current larger than the dark current. Figure 4c exhibits the *I*_ds_ − *V*_ds_ curves with and without laser illumination (2.8 μm, 37.1 μW; 4.2 μm, 40.6 μW; 10 μm, 41.6 μW). Different from the photovoltaic effect, a photocurrent is generated at forward bias. In addition, the photocurrent increases with *V*_ds_ and shows no indication of saturation, which further confirms the bolometric mechanism. The photocurrent under 10 μm laser illumination is larger than that at the other two wavelengths, which can be ascribed to the reflectivity of VO_2_ at 10 μm being smaller than that at 2.8 and 4.2 μm^49^. In other words, the device is more suitable for detecting LWIR. The responsivity is calculated at different forward biases, as shown in the inset of Fig. 4c. The responsivity under 2.8 μm laser illumination is slightly larger than that under 4.2 μm when *V*_ds_ < 0.2 V, which we think is due to the electronic transition of impurities and defect bands in the polycrystalline thin film, as confirmed by the absorption spectrum in Fig. S6b.

Figure 4d shows the time-resolved response at *V*_ds_ = 1 V under laser illumination of three wavelengths. The currents show an obvious contrast with and without laser illumination, although the rise and fall times are slightly long, which is determined by the heat capacity of the device and substrate. Effective methods can be used to enhance the performance, such as thinning the substrate thickness and introducing a suspended support^50^. The power dependence is investigated by measuring *I*_ds_ − *V*_ds_ curves under different laser powers, as shown in Fig. S9. Figure S9 also exhibits the |*I*_ds_| − *V*_ds_ curves on a logarithmic ordinate, in which the current at reverse bias is also larger than the dark current. This occurs because the thermal equilibrium is influenced as well as the Fermi level, with the result being an enlarged reverse saturation current under laser illumination. Figure 4e shows the calculated responsivity at different powers. The responsivity for each wavelength remains almost constant, which indicates the stability of our device. However, the responsivity suddenly increases from 0.06 to 0.47 A/W at 157 μW. This drastic change occurs during the MIT because the absorbed laser energy exceeds the energy for VO_2_ to undergo a transition from a semiconductor to a metal. The time-resolved photoresponse under 157 μW of 4.2 μm light is shown in Fig. 4f. There are two stages in the increase in the current, corresponding to the slopes in the inset of Fig. 4a. *I*_ds_ increases slowly at first and then increases rapidly when the absorbed energy reaches the energy required for the phase transition. This abrupt increase in current also indicates the transformation of our device from the p–n junction to the Schottky junction. Finally, the current tends to saturate because VO_2_ has completely changed into a metal, and its electric resistance changes little under laser illumination. When the laser is removed, the current rapidly decreases due to the rapid phase transition of VO_2_. Based on this phenomenon, the device can also be used for the detection of ultrahigh laser power.

### Schottky junction mode

**Fig. 5:**
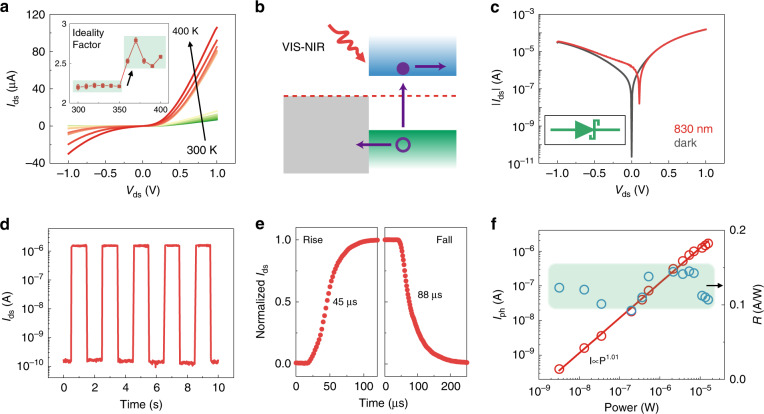
Schottky junction mode of the heterostructure. **a** *I*_ds_ − *V*_ds_ curves under temperatures from 300 to 400 K. The inset shows the ideality factor as a function of temperature. **b** Schematic of the photovoltaic mechanism of the Schottky junction for detecting VIS to NIR light. **c** *I*_ds_ − *V*_ds_ curves on a logarithmic scale under the dark state and 830 nm laser illumination. **d** Temporal response of the photocurrent under laser illumination at 830 nm; the power is 15.7 μW. **e** Rise and fall times of the normalized photocurrent under zero bias measured by an oscilloscope. **f** *I*_ph_ and responsivity as a function of the incident laser power.

As discussed above, the device transforms to a Schottky junction when the laser power exceeds the MIT energy. Here, the process of the mode changing with temperature and the performance of the Schottky junction mode are comprehensively investigated. Figure 5a shows the *I*_ds_ − *V*_ds_ curves from 300 to 400 K. The current rapidly increases from 350 to 360 K, indicating the mode switching of the device. The inset shows the calculated ideality factor *n*, which suddenly changes from 2.2 to 2.5, corresponding to the p–n junction transforming to the Schottky junction. In the Schottky junction, electrons diffuse from MoTe_2_ to the interface when the Fermi levels are equalized. The SCR appears on the side of the semiconductor, forming a built-in electric field directed from MoTe_2_ to the surface of VO_2_. Under laser illumination, the electrons of MoTe_2_ can be excited to the valance band, leaving an electron–hole pair. The electron–hole pair is then separated by the built-in electric field (Fig. 5b), which seems similar to the p–n junction at ambient temperature. However, as VO_2_ has completely transformed into a metal, the cutoff wavelength is determined by the bandgap of MoTe_2_ rather than VO_2_. Figure 5c presents the |*I*_ds_| − *V*_ds_ curves with and without laser illumination (830 nm, 15.7 μW) at 400 K. The reverse dark current increases with the bias due to the lowering of the barrier, which is the significant difference with the p–n junction. Figure 5d shows the temporal response under laser illumination in which the dark current increases from 0.2 pA to 0.2 nA. The increased dark current seems to degrade the performance of the photodetector, however, the significant on/off ratio as large as 10^4^ makes it still able to function as a photodetector. As shown in Fig. 5e, the rise and fall times are measured and evaluated as 45 and 88 μs, respectively, which are longer than those in the p–n junction mode. The performance of the device for different illumination intensities is exhibited in Fig. S10. It can be seen that *I*_sc_ significantly increases, especially in logarithmic coordinates, at zero bias. The *V*_oc_ values extracted from *I*_ds_ − *V*_ds_ curves are plotted and fitted as a function of log(*P*). The interlayer recombination order *β* is calculated to be 2.57, indicating a bimolecular recombination process, different from the monomolecular process in the above p–n junction. The power dependence and responsivity are further investigated at 400 K. *I*_ph_ scales linearly with power, and no evidence of saturation is observed in the measured range. The responsivity is calculated to be ~0.12 A/W. The reduced performance results from the photons only being absorbed by MoTe_2_, while metallic VO_2_ contributes nothing. Due to the metallization of VO_2_, EQE also decreases to ~18% (Fig. S10). The relatively outstanding performance under high temperature is definitely a breakthrough in the infrared detector.

## Discussion

A unique heterostructure based on VO_2_ and MoTe_2_ is demonstrated. As self-synthesized VO_2_ behaves as a p-type semiconductor and thin MoTe_2_ behaves as an n-type material, the heterostructure is a perfect p–n junction that acts as an ultrasensitive and ultrafast detector. A forward bias makes it switch to bolometer mode, capable of detecting broadband radiation from MWIR to LWIR. In addition, high temperatures above *T*_*c*_ lead to the transformation of VO_2_ from a semiconductor to a metal, resulting in the third operating mode of the Schottky junction, which is stable under extreme conditions with prominent performance. The heterostructure takes advantage of the photovoltaic effect and bolometric effect, exhibiting multiple functions covering the visible to LWIR, which is a promising approach for advanced uncooled photodetectors.

## Materials and methods

### Materials and characterization

The VO_2_ film was synthesized by direct current magnetron sputtering and thermal oxidation. First, a metal vanadium thin film was deposited under an Ar atmosphere of 1.2 × 10^−1^ Pa. The obtained metal vanadium film was then put into an annealing furnace for oxidation in a mixture gas of Ar and air (70 sccm) at 470 °C.

MoTe_2_ was purchased from 2D Semiconductors Inc. and thinned by mechanical exfoliation.

All the morphological structures of the devices were characterized by a Nikon optical microscope. AFM images were taken by a Bruker Dimension Edge in tapping mode. XRD measurements were performed by using a Bruker D8 Discover. The Raman spectra were obtained by a Lab Ram HR800 from HORIBA with a 514 nm excitation laser. The TEM images and EDS mapping were obtained by a JEOL JEM2100F TEM with an EX-24063JGT EDS.

### Device fabrication and measurements

The heterostructure devices were fabricated by restacking techniques. First, the VO_2_ film was patterned by UV photolithography and Ar plasma etching. MoTe_2_ flakes were exfoliated by scotch tape onto polydimethylsiloxane and transferred to the top of etched VO_2_ by dry transfer^12^. Polymethyl methacrylate and conductive ink were spin-coated (4000 rpm for 45 s) on the substrate for electron beam lithography to define electrodes. Pt/Au (20/60 nm) was sputtered as electrodes on MoTe_2_ and VO_2_ to form ohmic contacts. After the lift-off process, a heterostructure device was obtained.

The electronic and temperature dependence measurements of VO_2_ were performed by using an Agilent B2902A in a vacuum chamber probe station with a temperature control unit (K2000, MMR Technologies, Inc.). The electronic and optoelectronic measurements from 450 nm to 2 μm were conducted using a Keysight B1500A semiconductor parameter analyzer on a Lake Shore probe station. The response time data were acquired from a high-speed Tektronix (Tektronix MDO3014). The optoelectronic measurements at 2.8, 4.2, and 10 μm and scanning photocurrent microscopy were performed with a homemade optoelectronic measurement system.

## Supplementary information

Supplementary Information for A Versatile Photodetector Assistted by Photovoltaic and Bolometric Effects
