# Supplementary material for: A versatile photodetector assisted by photovoltaic and bolometric effects
Source: Light Sci Appl. 2020 Sep 10;9:160. doi: 10.1038/s41377-020-00396-3 (PMC7484767; doi:10.1038/s41377-020-00396-3)
Supplement: Supplementary file 1 — Supplementary Information for A Versatile Photodetector Assistted by Photovoltaic and Bolometric Effects [file 41377_2020_396_MOESM1_ESM.docx]

**Supplementary Information for**

**A Versatile Photodetector Assisted by Photovoltaic and Bolometric Effects**

*Wei Jiang,^1,2^ Tan Zheng,^3^ Binmin Wu,^1^ Hanxue Jiao, ^1^Xudong Wang,^1^ Yan Chen,^1^ Xiaoyu Zhang,* ^1^ *Meng Peng, ^1^ Hailu Wang, ^1,^*^2^ *Tie Lin, ^1^ Hong Shen, ^1^ Jun Ge, ^1^ Weida Hu^1*^, Xiaofeng Xu, ^3*^ Xiangjian Meng, ^1^ Junhao Chu, ^1^ Jianlu Wang^1*^*

^1^ State Key Laboratory of Infrared Physics, Shanghai Institute of Technical Physics, Chinese Academy of Sciences, 500 Yutian Road, Shanghai 200083, China

^2^ University of Chinese Academy of Sciences, Beijing, China

^3^ Department of Applied Physics, Donghua University, No. 2999, North Renmin Road, Songjiang District, Shanghai, 201620, China

*Corresponding author

E-mail: jlwang@mail.sitp.ac.cn, xxf@dhu.edu.cn, wdhu@mail.sitp.ac.cn


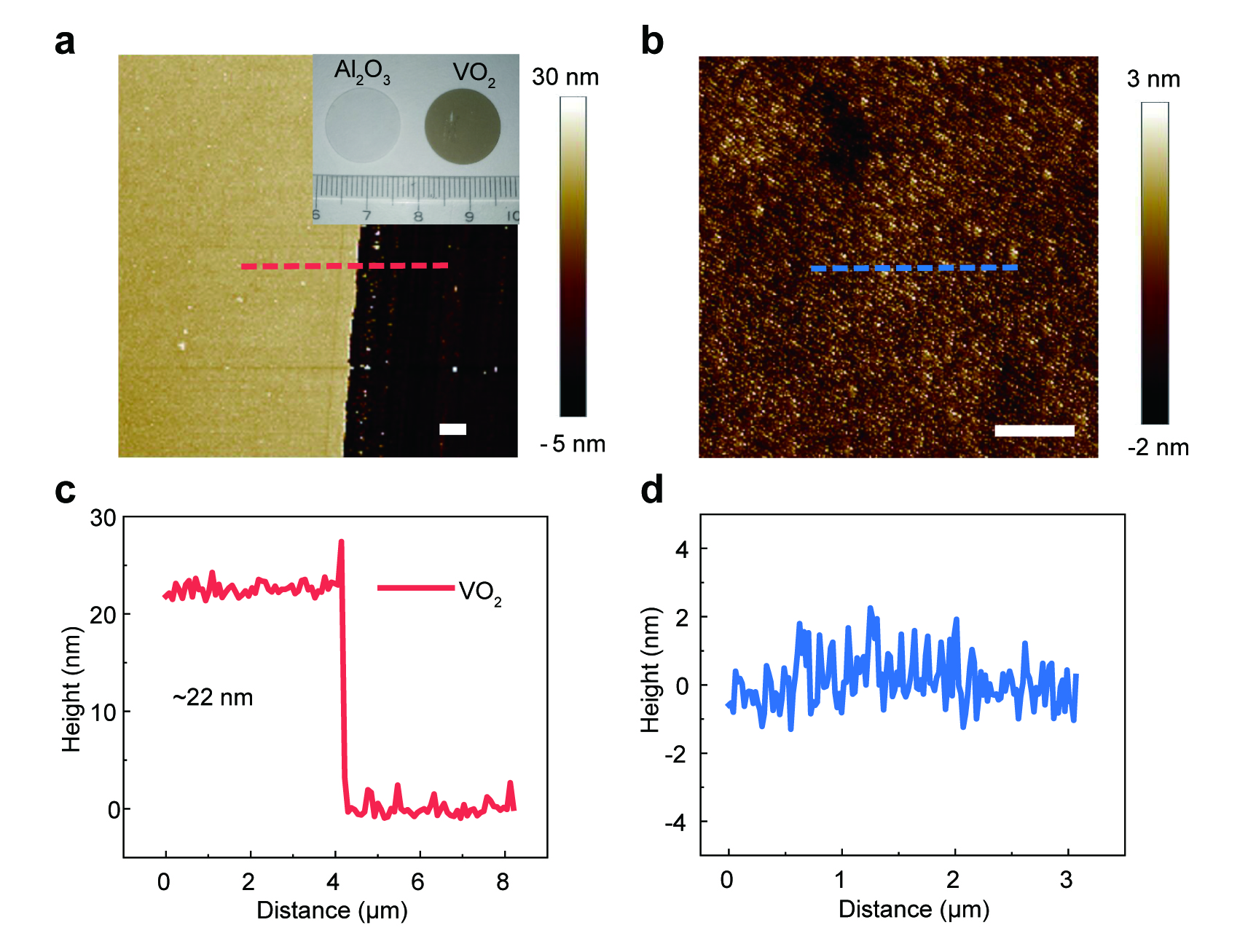


**Figure S1| a) b)** AFM image of synthesized VO_2_ film on the sapphire substrate. Inset in **a)** is the contrast optical image of sapphire with and without VO_2_ film. Scale bar, 1 μm. **c) d)** The height profile of the VO_2_ film marked in **a) and b)**. The thickness is about 22 nm with surface undulation less than 2 nm.


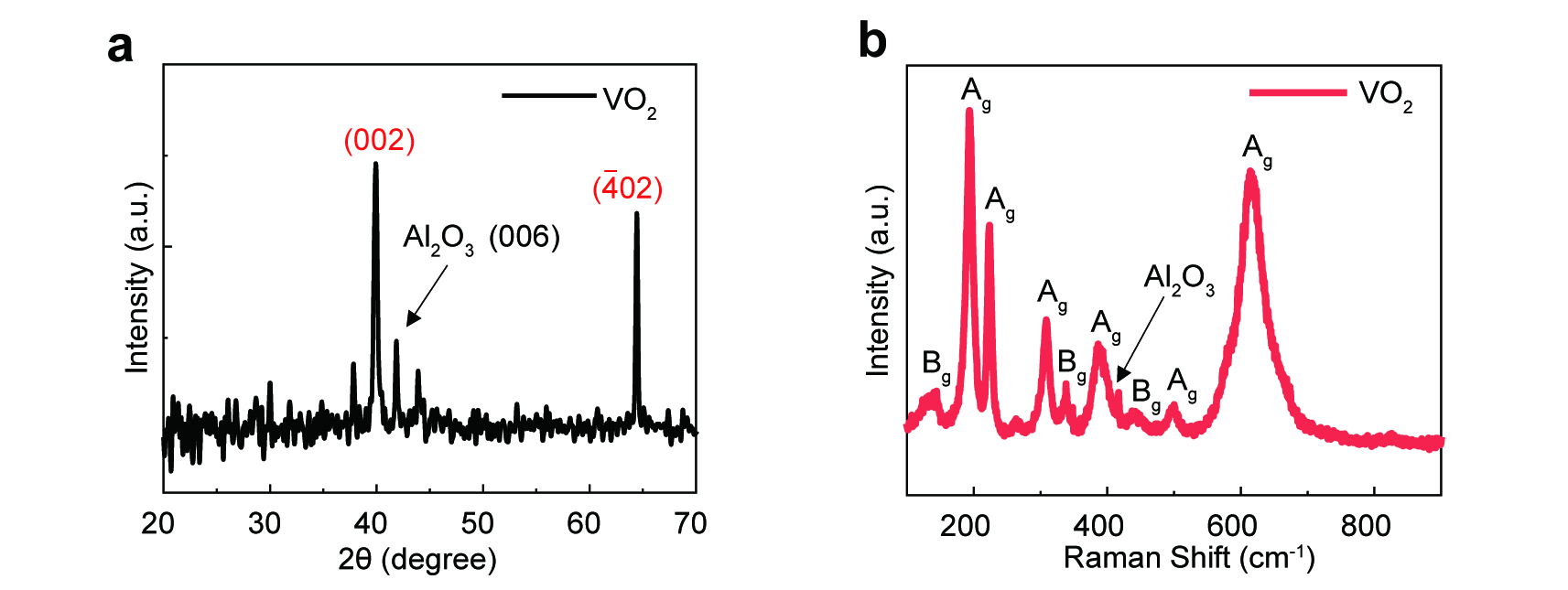


**Figure S2| a) b)** XRD and Raman spectrum of VO_2_ at ambient temperature. The diffraction peaks in XRD and vibration peaks in Raman prove its pure phase and monoclinic structure.


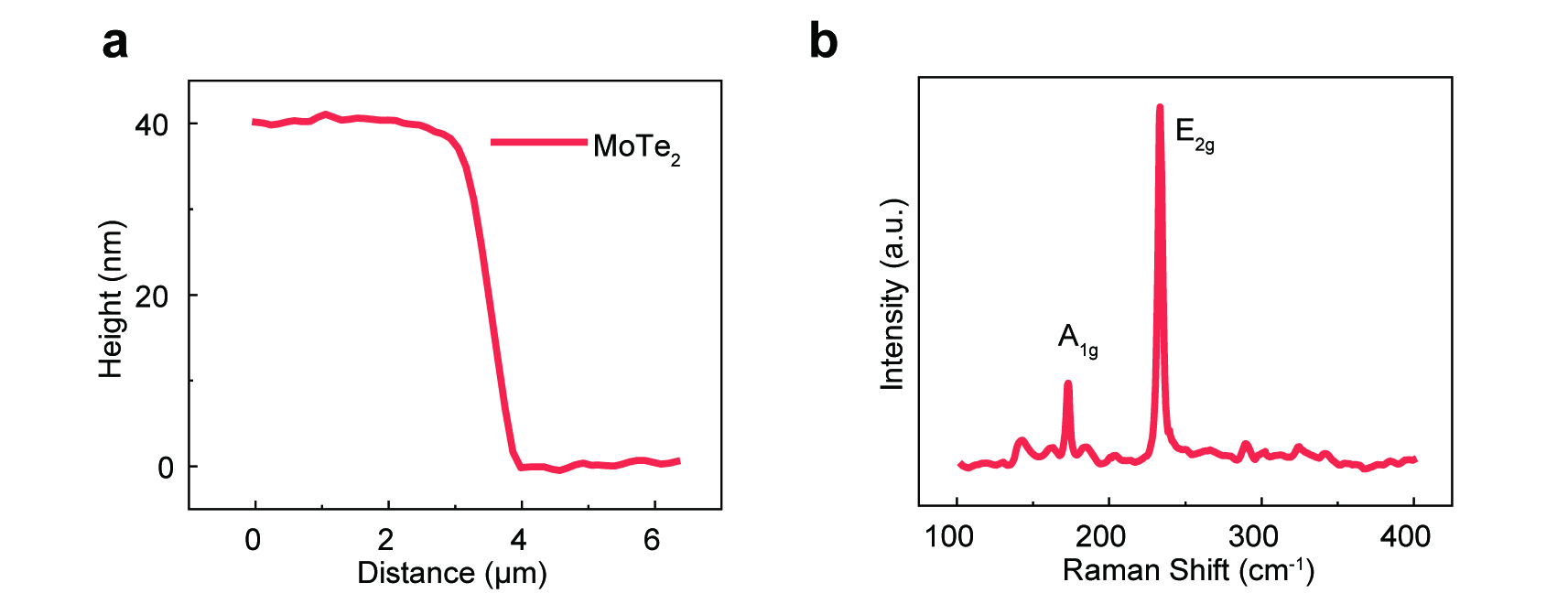


**Figure S3| a)** Height profile of MoTe_2_ in Fig 2b. The thickness is about 40 nm. **b)** Raman spectrum of MoTe_2_, two significant peaks at 173 cm^-1^ and 233^-1^ corresponding to the A_1g_ and E_2g_ vibration modes of MoTe_2_.


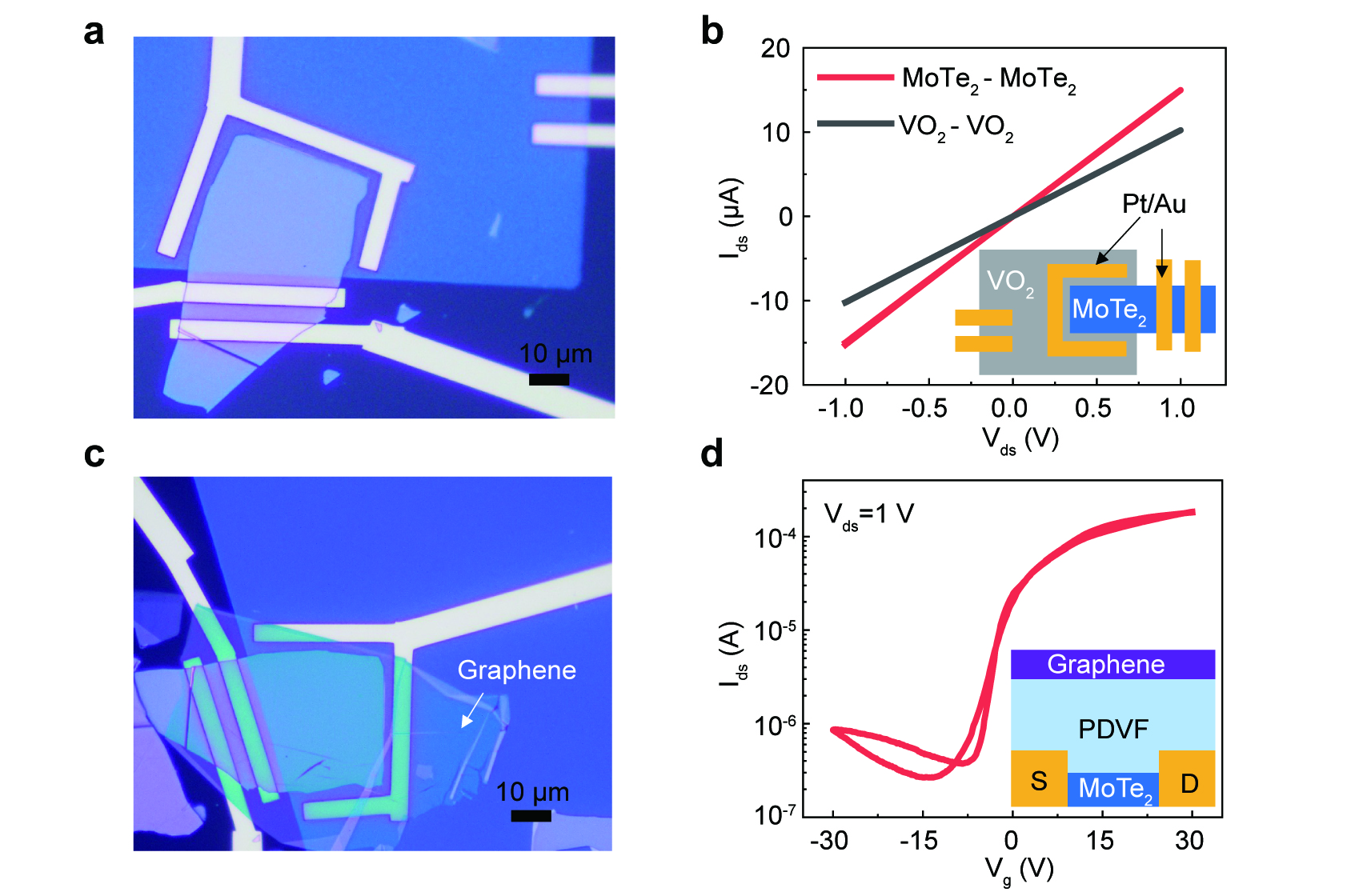


**Figure S4| a)** Optical image of MoTe_2_/VO_2_ heterostructure. **b)** Output characteristic of single MoTe_2_ flake and VO_2_ film with Pt/Au electrodes. The contacts are both show ohmic behavior. **c)** Optical image MoTe_2_ FET with P(VDF-TrFE) as dielectric layer and graphene as top gate. d) Transfer characteristic of MoTe_2_ FET. Our MoTe_2_ in heterostructure is a n-type semiconductor.


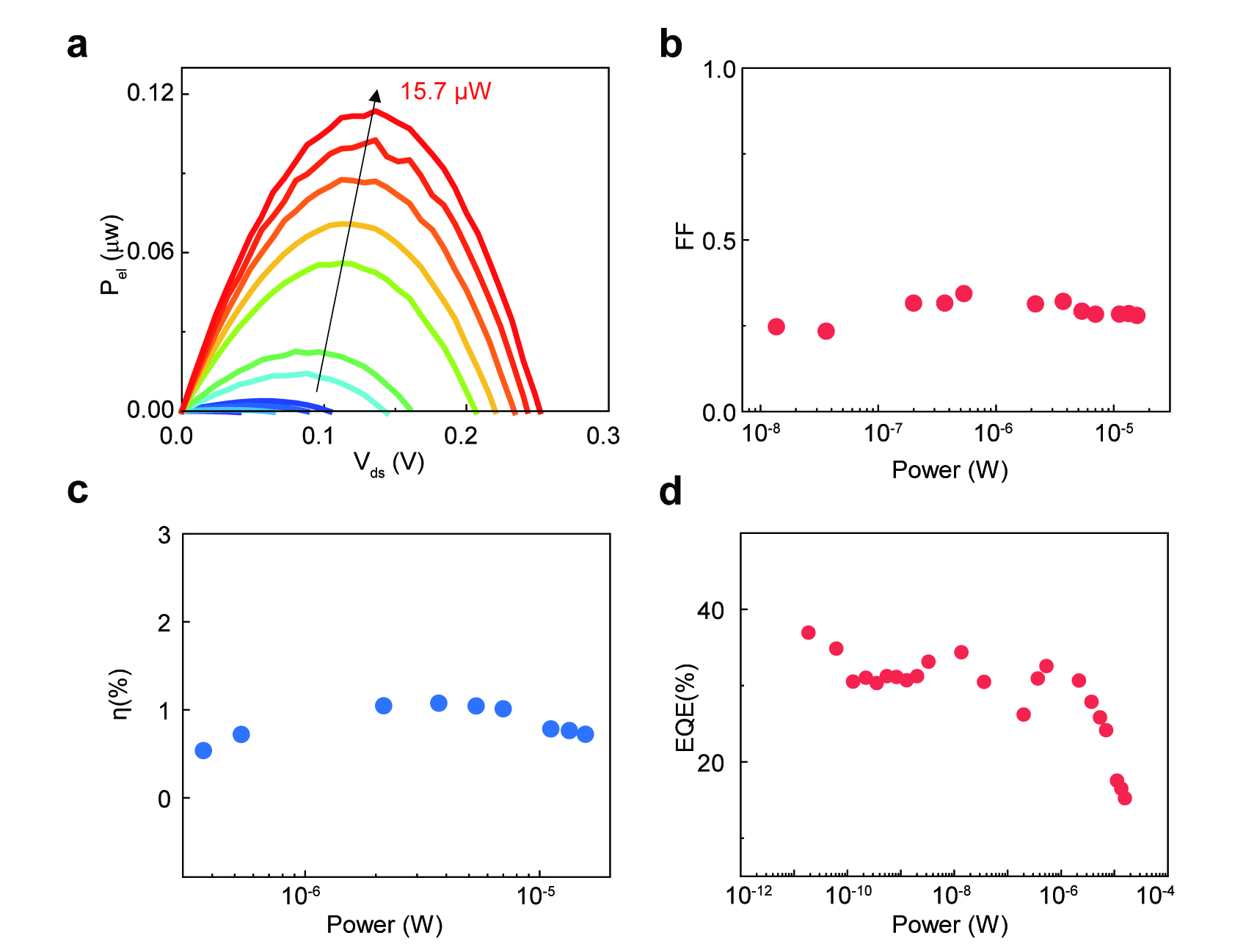


**Figure S5| a)** The output electric power as a function of V_ds_. **b) c) d)** The fill factor, power conversion efficiency and external quantum efficiency extracted from fabricated MoTe_2_/VO_2_ heterostructure.


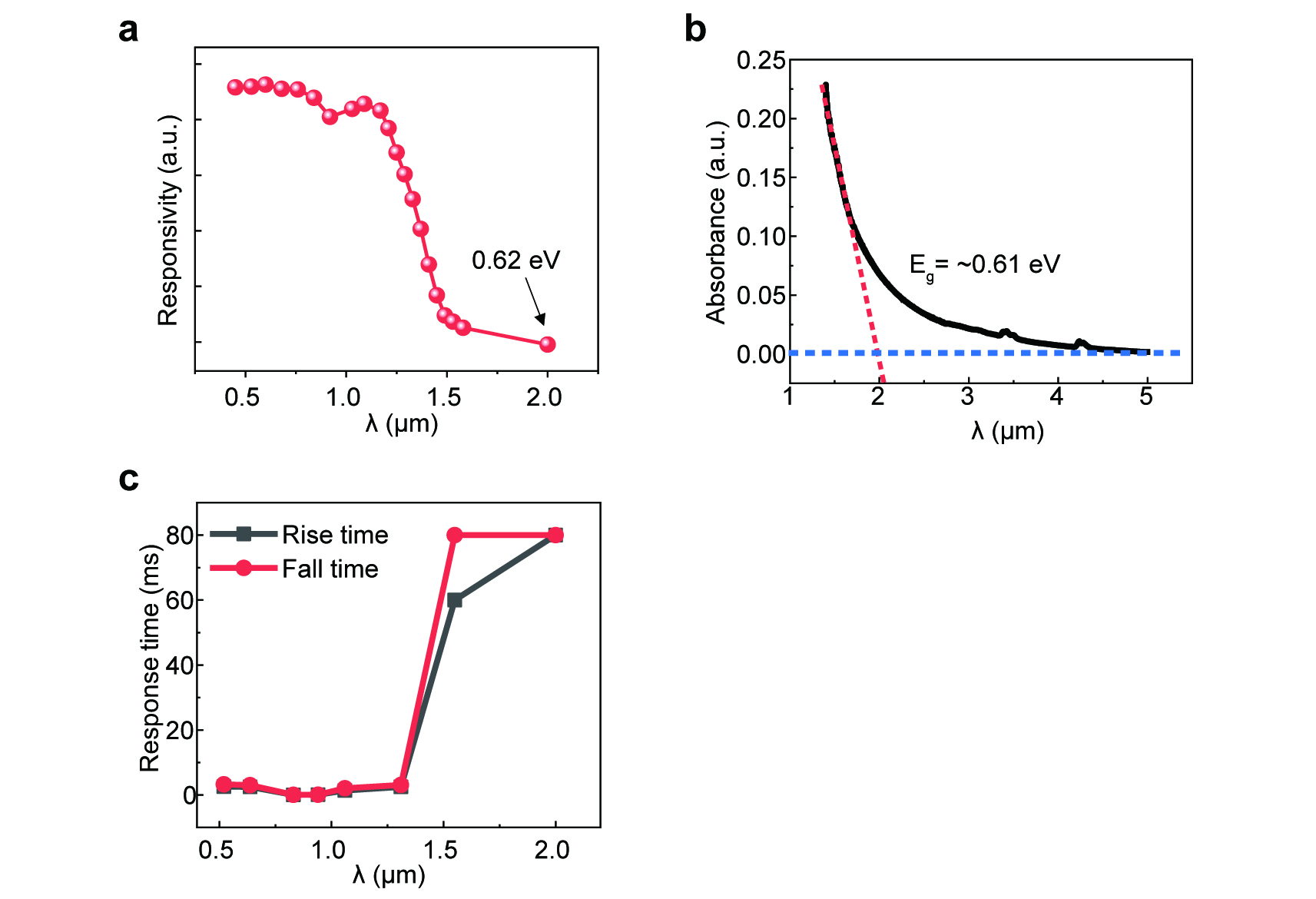


**Figure S6| a)** the responsivity of our heterostructure under the radiation from 0.45 μm and 2 μm. **b)** the absorbance of VO_2_ film from 1.4 μm to 5 μm. **c)** the response time as a function of wavelength from 0.45 μm and 2 μm.


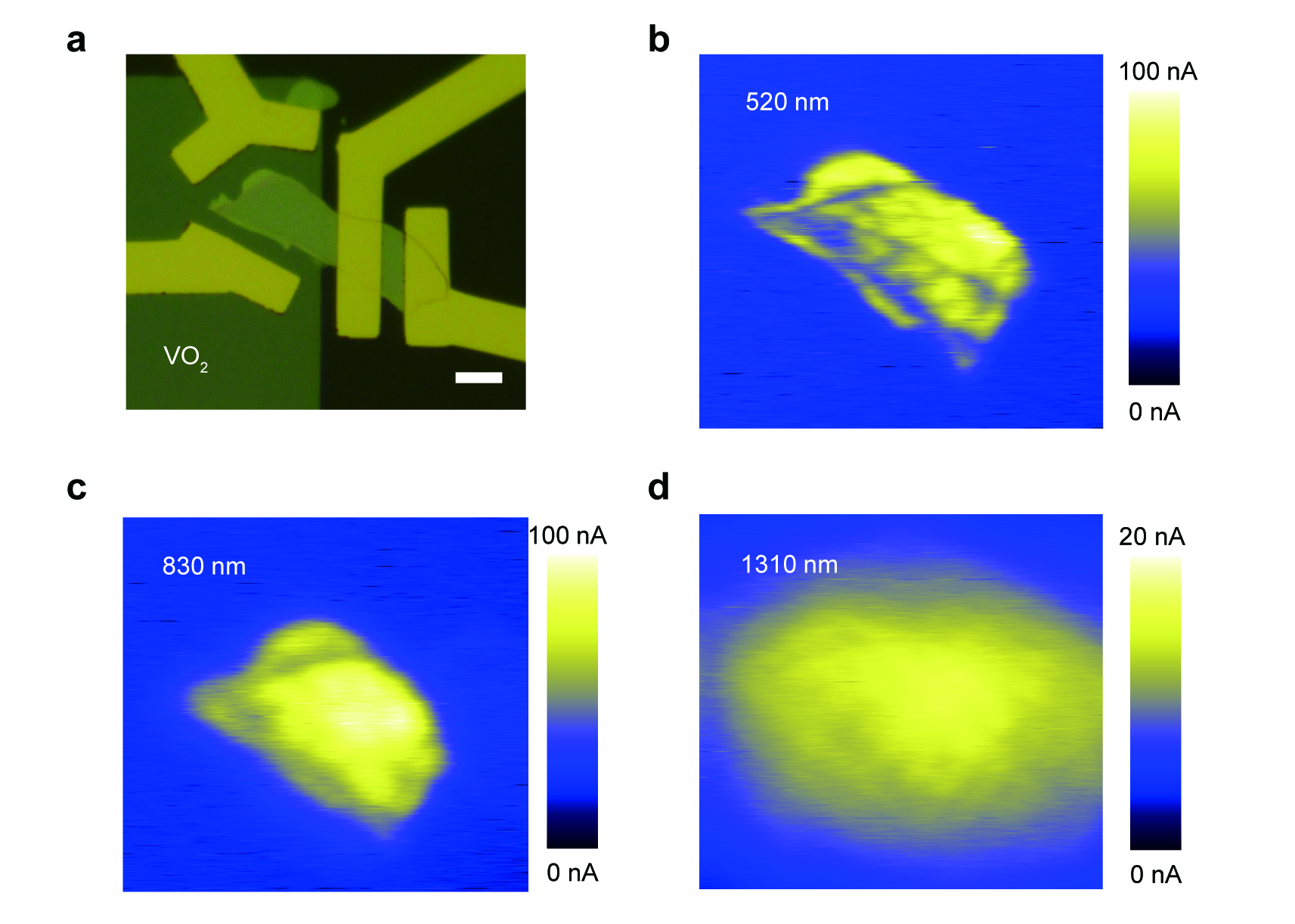


**Figure S7| a)** Optical image of device 2^th^. Scale bar, 10 μm. **b) c) d)** Scanning photocurrent microscopy of 520 nm, 830 nm and 1310 nm.


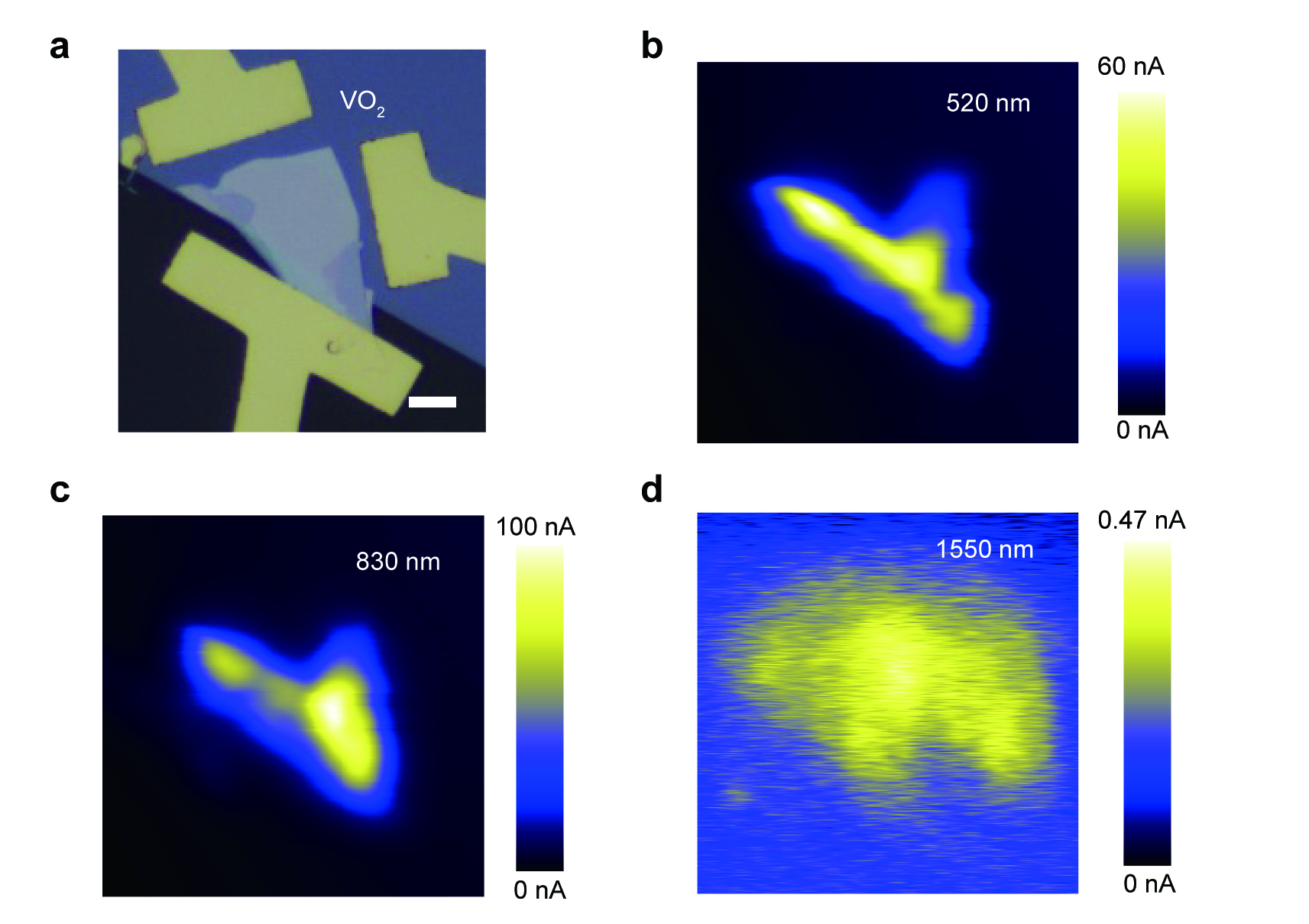


**Figure S8| a)** Optical image of device 3^th^. Scale bar, 10 μm. **b) c) d)** Scanning photocurrent microscopy of 520 nm, 830 nm and 1550 nm.


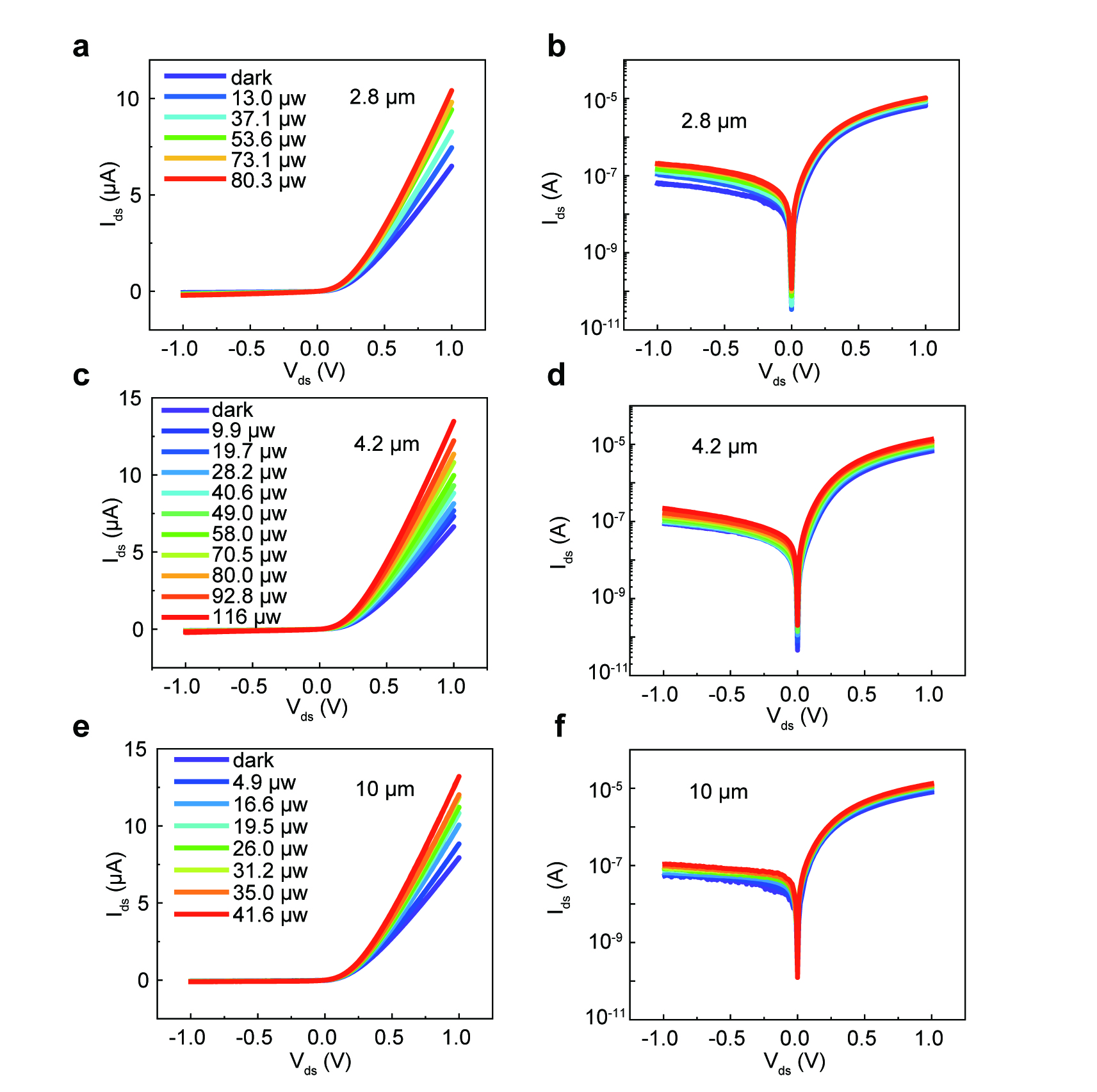


**Figure S9|** The power-dependence of the device with bolometric mode at 2.8 μm (**a** and **b**), 4.2 μm (**c** and **d**) and 10 μm (**e** and **f**) in liner and logarithmic scale.


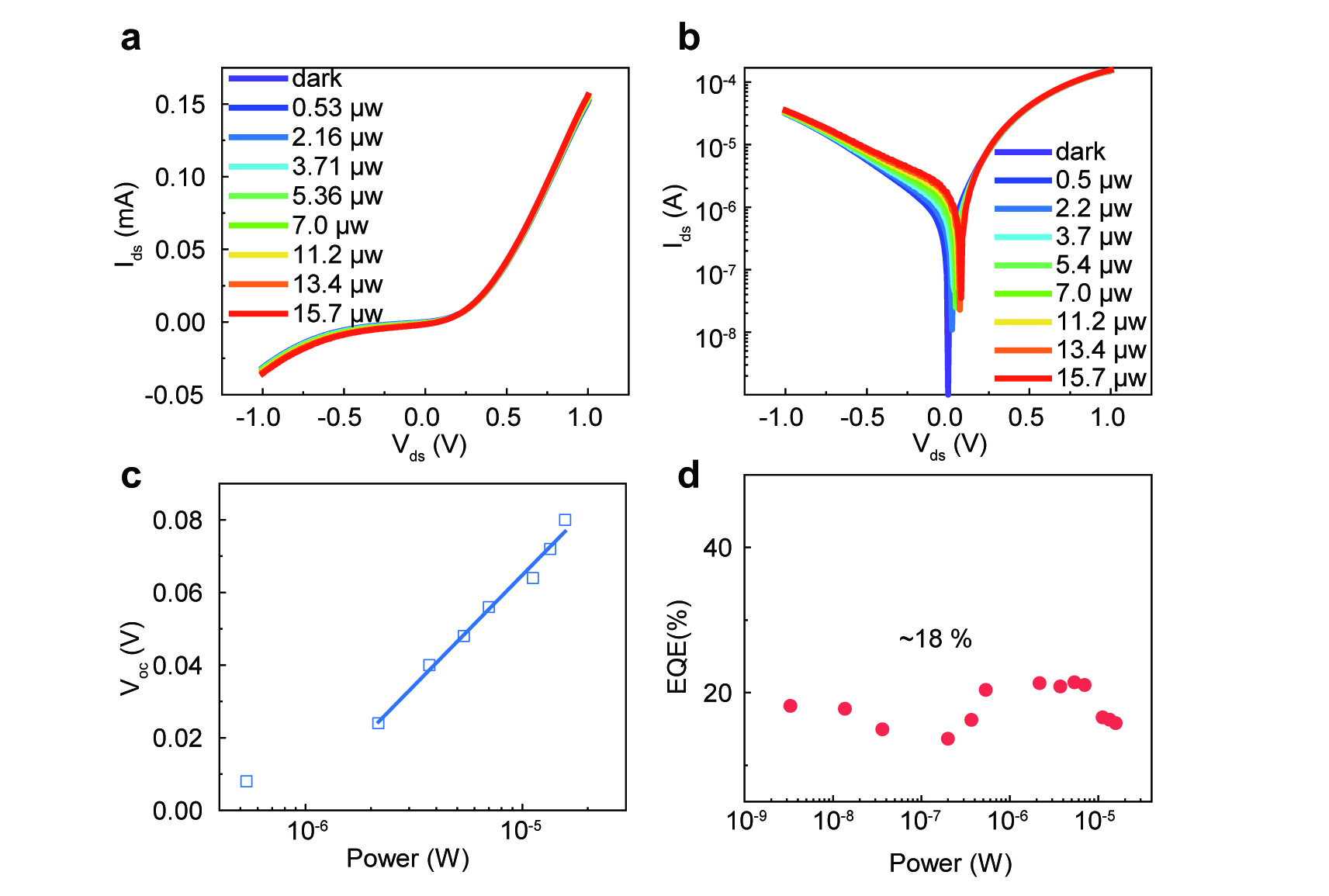


**Figure S10| a) b)** The power-dependence of the device with Schottky mode in liner and logarithmic scale. **c)** V_oc_ as a function of power. the **d)** External quantum efficiency of Schottky mode
